# A Novel 90-kbp Deletion of *RUNX2* Associated with Cleidocranial Dysplasia

**DOI:** 10.3390/genes13071128

**Published:** 2022-06-23

**Authors:** Yanli Zhang, Xiaohong Duan

**Affiliations:** State Key Laboratory of Military Stomatology, National Clinical Research Center for Oral Diseases, Shaanxi Key Laboratory of Stomatology, Department of Oral Biology, School of Stomatology, The Fourth Military Medical University, Xi’an 710032, China; swzhangyanli@126.com

**Keywords:** cleidocranial dysplasia, large fragment deletion, *RUNX2*, novel mutation

## Abstract

Cleidocranial dysplasia (CCD) is a rare autosomal dominant skeletal dysplasia caused by runt-related transcription factor 2 (*RUNX2*) mutations. In addition to the regular missense, small or large fragment deletions are the common mutation types of *RUNX2*. This study aimed to find the rules of deletions in *RUNX2*. The clinical information of one Chinese CCD family was collected. Genomic DNA was extracted for whole-exome sequencing (WES). Bioinformatics analyzed the pathogenicity of the variants. Polymerase chain reaction (PCR) and Sanger sequencing were carried out using specific primers. RT-PCR and Q-PCR were also used to detect the mRNA level of *RUNX2*. The CCD studies related with deletions in *RUNX2* from 1999 to 2021 from HGMD and PubMed were collected and analyzed for the relationship between the phenotypes and the length of deleted fragments. The proband presented typical CCD features, including delayed closure of cranial sutures, clavicle dysplasia, abnormal teeth. WES, PCR with specific primers and Sanger sequencing revealed a novel heterozygous 90-kbp deletion in *RUNX2* (NG_008020.2 g.103671~193943), which caused a substitution (p.Asn183Ile) and premature termination (p.Asp184*). In addition, the mRNA expression of *RUNX2* was decreased by 75.5% in the proband. Herein, 31 types of deletions varying from 2 bp to 800 kbp or covering the whole gene of *RUNX2* were compared and the significant phenotypic difference was not found among these deletions. The CCD phenotypes were related with the final effects of *RUNX2* mutation instead of the length of deletion. WES has the defects in identifying large indels, and direct PCR with specific primers and Sanger sequencing could make up for the shortcoming.

## 1. Introduction

Cleidocranial dysplasia (CCD; MIM 119600) is a rare hereditary disease. It exhibits delayed closing or non-closing of the fontanels, aplastic or hypoplastic clavicles, and multiple dental anomalies. The reported dental malformations of CCD include retained deciduous teeth or delayed eruption of permanent and supernumerary teeth [1,2]. The phenotypic spectrum of CCD may be varied. CCD exhibits autosomal dominant heredity with an approximate prevalence of 1:1,000,000 [3].

CCD has been ascribed to the mutations in the runt-related transcription factor 2 (*RUNX2*) gene, which plays a critical role in bone metabolism by regulating the differentiation between osteoblasts and osteoclasts [4]. RUNX2 protein contains a highly conserved Runt domain [5,6]. Reportedly, the common mutations in *RUNX2* included chromosomal translocations and deletions, insertion, deletion, missense, nonsense, splice-site, and frameshift mutations [7]. To date, 200 heterozygous mutations in *RUNX2* have been identified in individuals with CCD (Human Gene Mutation Database, HGMD, www.hgmd.cf.ac.uk, accessed on 29 April 2022) [6,8]. The majority of *RUNX2* mutations were missense, primarily in the Runt domain [7]. Approximately 13% of the reported CCD cases have microdeletions in *RUNX2* [9]. Whether the large deletion mutation has a more significant impact on the function of the *RUNX2* gene compared to microdeletions and missense mutation is yet unknown. In order to elucidate the characteristics of a large deletion mutation in *RUNX2* with CCD. Herein, we reported a Chinese CCD family with a novel 90-kbp deletion and summarized the reported deletions of the *RUNX2* gene in HGMD and literature.

## 2. Materials and Methods

### 2.1. Clinical Manifestations

The proband was an 18-year-old male, referred to the Clinic of Oral Rare Diseases and Genetic Diseases, School of Stomatology at the Fourth Military Medical University, with the chief complaint of retention of deciduous teeth and abnormal facial features. The patient, his mother and his sister were examined and evaluated with panoramic radiographs or computed tomography (CT). This study was approved by the Ethics Committee of the School of Stomatology, The Fourth Military Medical University (approval no. 2018-014). Informed consent was obtained from all the participants in this study. 

### 2.2. Whole-Exome Sequencing (WES) and Identification of Variants

Genomic DNA (gDNA) was extracted from peripheral blood samples of patients with CCD and his family members using QIAamp DNA Blood Mini kit (Qiagen, Valencia, CA, USA), according to the manufacturer’s instructions. WES containing exome capture, high-through put sequencing, and common filtering was performed using Annoroad Gene Technology (Annoroad, Beijing, China). Alignment of the sequence reads, indexing of the reference genome, variant calling, and annotation were carried out using the Agilent SureSelect Human All Exon V6 system (Agilent Technologies, Santa Clara, CA, USA). Valid sequencing data of WES are mapped to the human reference genome GRCh37 using the Maq program. 

Six pathogenicity prediction software (SIFT, PolyPhen-2 HVAR, PolyPhen-2 HDIV, MutationAssesor, MutationTaster, and CADD) [10] were used for mutation identification as likely damaging, deleterious, and disease causing; due to the possibility of amino acid alternation, the nonsense or frameshift (insertion or deletion) mutation was considered; the variants occurring in exon or splice sites and associated with CCD or involved in bone development were prioritized. All variants not relevant to known phenotypes or inheritance patterns were excluded; the variant was previously described as disease-causing in the HGMD or in the open published literature. 

### 2.3. Polymerase Chain Reaction (PCR) and Sanger Sequencing

WES and bioinformatic analysis identified *RUNX2* as a pathogenic gene of the family, then disease-associated *RUNX2* mutation was further confirmed by PCR using two pairs of specific primers for *RUNX2* (NM_001024630.4) (Pair 1: forward (F): 5′-GGCCATTACTGGACTGGACT-3′, reverse (R1): 5′-TCATCAAAGGAGCCTAATGTGCT-3′; Pair 2: forward (F): same as pair 1, reverse (R2): 5′-TAGGGCTAGTACTATAATGTAAC-3′). PCR was performed using 1 μg of template gDNA, 0.5 μL of each primer (10 μM), and 10 μL Taq PCR MasterMix (TaKaRa, Shiga, Japan) in a reaction volume of 20 μL. The amplification reactions were as follows 95 °C for 5 min, followed by 35 cycles of 95 °C for 1 min, 58 °C for 30 s, and 72 °C for 1 min, with a final extension of 72 °C for 7 min. The PCR products were visualized by 1% agarose gel electrophoresis and analyzed by Sanger sequencing on an ABI 3500 automated sequencer (Applied Biosystems, Thermo Fisher Scientific, Waltham, MA, USA). Sequence data were analyzed using Sequencher software (version 5.0; Gene Codes Co., Ann Arbor, MI, USA).

### 2.4. Quantitative PCR Analysis (Q-PCR)

To validate the effect of the mutation, we performed Q-PCR and Reverse Transcription-polymerase chain reaction (RT-PCR) assays to determine the mRNA level of the *RUNX2*. According to the manufacturer’s instructions, total RNA was extracted from 293 cells using Trizol^TM^ reagent (Life Technologies, Carlsbad, CA, USA), or from whole blood samples of the proband and his family members using RNAprep pure Blood kit (Tiangen, Beijing, China). The concentration of the isolated RNA was determined by Thermo Scientific NanoDrop 2000/2000c spectrophotometer. cDNA was reverse transcribed using PrimeScript^TM^ RT Master Mix (Takara, Dalian, China). The third pair of *RUNX2* primers for Q-PCR or RT-PCR were as follows: forward (F3): 5′-TCATGGCGGGTAACGATGAA-3′, reverse (R3): 5′-TCCCGAGGTCCATCTACTGT-3′. Q-PCR was performed in a reaction volume of 20 μL containing 10 μL of SYBR Premix Ex Taq (TaKaRa), 100 ng cDNA, and 0.2 μL of each primer (10 μM). The reaction was as follows: 95 °C for 5 min and 40 cycles of 95 °C for 5 s, 62 °C for 15 s, and 72 °C for 15 s. The data were normalized to that of the control cDNA from 293 cells. The relative expression levels were calculated using the ∆∆CT method.

### 2.5. Predictions of the Protein Structure

The mutant RUNX2 protein was analyzed using the SWISS-MODEL software. The known structure of the Runt domain of RUNX1 protein was used as the template of RUNX2.

### 2.6. Retrospective Study

We summarized the reported deletions of the *RUNX2* gene in HGMD and literature. The related CCD references were searched with respect to the large deletion in CCD patients. The following keywords were used to search the related references (1991 to present) from PubMed: cleidocranial dysplasia, deletion, and *RUNX2*. A total of 19 articles that matched the search criteria were retrieved from PubMed, and only those references describing the deletions of *RUNX2* were included in our analysis. Finally, 31 deletions varying from 2 bp to 800 kbp or covering the whole gene of *RUNX2* were included to summarize the general characteristics of deletion mutation in CCD with *RUNX2* mutation.

## 3. Results

### 3.1. Clinical Findings

**Figure 1:**
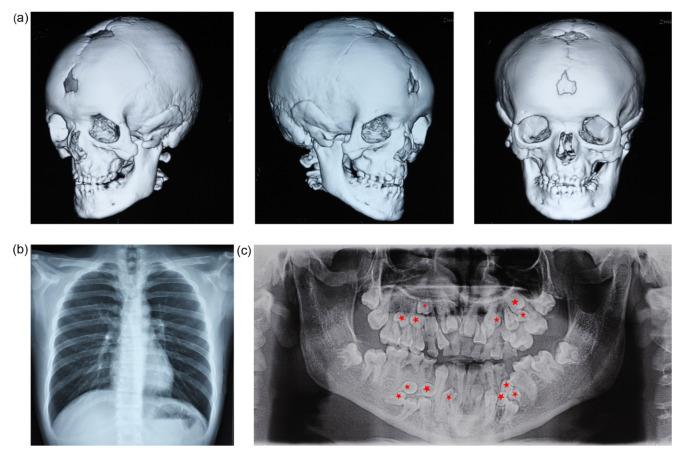
Clinical and radiographic findings of the proband. (**a**) Different views of CT images. The skull shows non-closing fontanel, frontal bossing, and malocculusion. (**b**) Chest radiograph demonstrates cone-shaped thorax and hypoplasia of clavicles. (**c**) Panoramicradiograph shows dental anomalies, including 13 supernumerary teeth (red stars), 14 retained deciduous teeth (51–53, 61–65, 72–74, 82, 84, 85), and 16 failed eruption of permanent teeth (11–13, 21–25, 32–35, 42, 44, 45, 47) (not including the third molars, which have to be followed up).

The proband (II_2_) was diagnosed with CCD. The patient showed typical CCD phenotypes: abnormal facial features, underdevelopment of the middle face, protrusion of mandible, and typical dental abnormalities of CCD such as retention of primary teeth (Figure 1). CT examination revealed remarkable open fontanelles, delayed closure of cranial sutures (Figure 1a), maxilla dysplasia, hypoplastic of clavicles, and multiple dental anomalies (Figure 1b). Panoramic radiograph showed retention of 14 deciduous teeth, failed eruption of 16 permanent teeth, and 13 supernumerary teeth (Figure 1c). No other signs of mental retardation or physical disability were observed in the patient. The mother (I_2_) and sister (II_1_) of the proband did not have any features associated with CCD.

### 3.2. Genetic Findings

After filtering and basic bioinformatic analysis of the WES raw data of the proband and his mother and sister, a heterozygous mutation in the *RUNX2* gene was revealed. A speculated 68 bp insertion mutation (c.547_548ins) in exon 4 of *RUNX2* (NM_001024630) was reported in the proband, which was not found in the non-affected members of the family.

### 3.3. PCR and Sanger Sequencing to Determine Mutation

**Figure 2:**
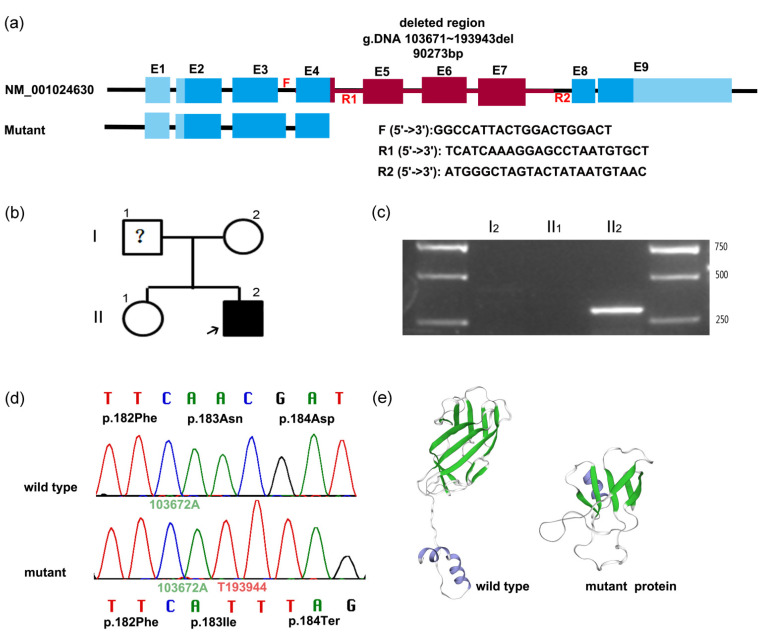
PCR and Sanger sequencing to determine mutation. (**a**) Schematic map of the deleted fragment and designed primer in *RUNX2* gene. Exons were labeled as solid rectangles in blue or red. The red zone between exon 4 and intron 7 represents a 90-kbp nucleotide detection. The first and second pairs of primers are F/R1 and F/R2, respectively. (**b**) Pedigree map. Arrow indicates the proband. The clinical phenotype and genotype of I_1_ is unknown and thus designated with question mark. (**c**) PCR result of *RUNX2* analysis. A 302-bp PCR product of the proband using the second pair of primers (F/R2) was amplified in the proband, not in other family members of I_2_ and II_1_, in which a 90574-bp fragment was supposed to be amplified. (**d**) Comparing the sequencing chromatograms around g.103672A (c.547A) of *RUNX2* of the proband. The upper one and lower one were from the PCR products amplified using the first pair of primers (F/R1), and the second pair of primers (F/R2), respectively. In the wild-type chain (**upper**), g.103673A (green) was next to g.103672A (green); while g.193944T (red) was followed by g.103672A (green) in the mutant chain (**lower**). (**e**) Predictive 3D structures of the wild-type and mutant RUNX2 proteins using SWISS-MODEL. Green ribbon represents the β-sheet, and the blue ribbon represents α-helix.

The first pair of primers were designed around c.547 of *RUNX2* (the forward (F) and reverse primer (R1) were in introns 3 and 4, respectively) (Figure 2a) and could result in 341 bp PCR products. Then the regular PCR was performed for the proband and his mother and sister. However, Sanger sequencing of PCR bands did not show any insertion or deletion in the proband (Figure 2d). Then we compared the sequence of the “inserted 68 bp” and found the 68 bp fragment was actually a part sequence of intron 7 of *RUNX2*. Thus, we assumed there might be a large deletion from exon 4 to intron 7. So we designed a new reverse primer (R2) in intron 7 (Figure 2a). Intriguingly, the proband had a positive band (Figure 2c) while other non-affected members did not amplify successfully due to the long distance between two primers of F and R2. The new PCR products (F/R2) of the proband were subjected to Sanger sequencing, and the exact breakpoints of the deletion were identified compared to the reference sequence of *RUNX2*. The 90,273 bp deletion lay between exon 4 and intron 7 (NG_008020.2 g.103671~193943) (Figure 2d), which was detected in the proband but not in the non-affected family members or the 50 unrelated healthy Chinese volunteers. Furthermore, this mutation has not been reported previously. 

### 3.4. Bioinformatics Analysis

The human *RUNX2* gene maps to chromosome 6p21 and consists of 8 exons encoding a 521-amino acid protein (NP_001019801.3). The novel deletion mutation causes a frameshift and subsequent premature translation termination. The Q184X truncating nonsense mutation generates a truncated 183-amino acid protein lacking a part of the Runt homologous domain (RHD), nuclear localization signal (NLS), and a part of the proline/serine/threonine rich region (PST). Additionally, we used SWISS-MODEL software to predict the effect of in-frame deletion on the overall protein structure. The three-dimensional (3D) structures of the wild-type and mutant RUNX2 proteins (g.103671–193943 del 90273) differed markedly. The novel deletion of 90-kbp in *RUNX2* produced a substantially truncated protein shorter than the wild-type protein, and α-helix and β-sheet were reduced in number and length (Figure 2e).

### 3.5. RUNX2 Expression Analysis

**Figure 3:**
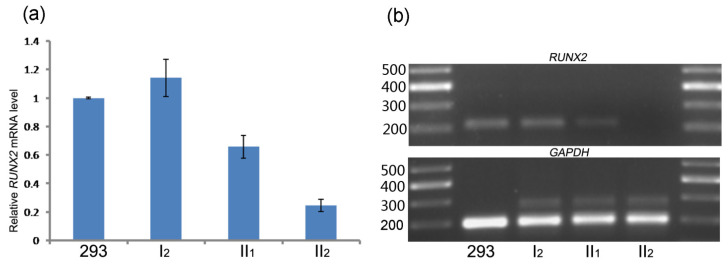
Comparison of *RUNX2* mRNA levels. (**a**) Q-PCR was performed to test the *RUNX2* expression levels. The proband (II_2_) carried about 24.5% normal *RUNX2*. (**b**) Regular RT-PCR assessed *RUNX2* mRNA levels. The *RUNX2* level of the proband was significantly down regulated compared to 293 cells, II_1_ and I_2_.

To further investigate the effect of the mutation, Q-PCR and regular RT-PCR were performed to determine the expression levels of *RUNX2*. The upstream and downstream primers were designed in exons 4 and 5, respectively. Q-PCR results indicated that the *RUNX2* levels were significantly down regulated in the proband compared to normal controls. The proband exhibited a 75.5% decrease in wild-type *RUNX2* levels resulting in CCD; and his mother and sister had the slight up (14.5%) and down (34.3%) changes comparing to the control (Figure 3a). RT-PCR analysis showed the similar results and the proband did not have an obvious band (Figure 3b).

## 4. Discussion

About 60–70% of the reported CCD cases with a clinical diagnosis harbor mutations in *RUNX2*. Currently, no other causative gene is indicated in CCD cases [11,12,13]. Hitherto, at least 200 *RUNX2* mutations have been reported in CCD patients [12,14]. The mutation types of *RUNX2* include missense, nonsense, frameshift, splicing, insertion, microdeletions, and microduplication (HGMD).

The microdeletions may be found in up to 10%–13% of the cases with CCD in *RUNX2* [15]. Approximately 2/19 (10%) of CCD cases may be caused by large chromosomal rearrangements that affect the *RUNX2* gene [16]. In this study, we identified a novel 90-kbp deletion (g.103671–193943 del 90273) between exon 4 and intron 7 in *RUNX2* in a Chinese CCD patient. In order to characterize the large deletion mutation in CCD with *RUNX2* mutation, we collected the reported deletions, large deletions, and translocation breakpoints, and summarized them in Table 1.

The variation in the deleting length of in *RUNX2* have not been compared previously. Fluorescence in situ hybridization (FISH) analysis, Q-PCR, or comparative genomic hybridization (CGH) microarray have been employed to identify these deletions, while PCR and Sanger sequencing were seldom used to elucidate the details of the deletion. In the current study, the WES reports from the company suggested a 68 bp insertion in *RUNX2* between c.547 and c.548, which could not be proved by us. On the contrary, we revealed that the “68 bp inserting fragment” was actually localized in intron 7 of *RUNX2* and finally identified a large deletion in the gene using a specific pair of primers and PCR. Therefore, WES has some defects in identifying large indels or other structural variations. PCR with specific primers and Sanger sequencing could help in elucidating the details of the deletion gap. Moreover, RT-PCR or Q-PCR are convenient and cost-effective supplementary tools to identify the indel effects on the mRNA expression.

Based on the retrospective analysis of large deletion of *RUNX2* with the described mutations details, we found that most of them result in a frameshift and premature translation termination, which had the same effects with point mutations causing premature translation termination. The large deletion mutation does not mean a more severe impact on the *RUNX2* function than the small deletions or missense mutations. Therefore, when the large fragment or even the entire gene of *RUNX2* is deleted, their pathogenic effects depend on the final amino acid changes, which in turn determine the related CCD phenotypes.

Type I *RUNX2* (MRIPV isoform; UniProt: Q13950-2) [32] and type II *RUNX2* (MASNS isoform; UniProt: Q13950-1) [33] are two isoforms with different spatiotemporal patterns. The promoter of type II *RUNX2* is termed “bone-related” because it drives the expression of the isoform widely in bones. The reference sequence of type II *RUNX2* mRNA (NM_001024630; NP_001019801.3) was used in the retrospective analysis of deletions. *RUNX2* is a multidomain protein containing glutamine–alanine repeats domain (QA), Runt homologous domain (RHD), nuclear localization signal (NLS), and the proline/serine/threonine rich region (PST) [30]. Most missense mutations of *RUNX2* uniquely occurred within the RHD region [30]. The missense mutations may prevent *RUNX2* binding to DNA, while the nonsense mutations may effectuate the biosynthesis of the truncated protein. The frameshift mutations are detected throughout the gene. The current retrospective study found that deletion mutations occurred throughout the *RUNX2* gene: 16.1% in the QA domain, 9.7% in the RHD region, 22.6% in the PST region, and 51.6% covered more than one domain.

The functional expressing level of *RUNX2* is required for the formation of bone. The threshold of normal *RUNX2* level to keep the normal bone development or causing CCD phenotypes was not clear. 79% of *Runx2*-deficient mice did not show the skeletal abnormalities due to harboring the partial wild-type *Runx2* transcripts [34]. Lou et al. reported that wild-type *Runx2* levels decreased to 70%, resulting in the CCD phenotype [34]. Another study reported that when the *RUNX2* activity declines to about 50%, the growth of the skeleton deteriorates and gradually worsens with further loss of gene activity in Japanese patients [5]. A mild CCD was reported as mosaicism with the deletion of exons 3–6 of *RUNX2*, who carried about 15% mosaic DNA [15]. In another study, the mother of one CCD patient did not exhibit any CCD phenotypes but carried about 21.8% mosaic DNA microdeletion in exons 1–4 of the *RUNX2* gene [29]. The current study found that the mRNA level of *RUNX2* in the proband (II_2_) was decreased by 75.5%, and significant CCD symptoms occurred. We also found that the mRNA level of *RUNX2* was reduced by 34.3% in the proband’s sister (II_1_) who did not have the *RUNX2* mutation and CCD symptoms. Therefore, it could be deduced that other factors may regulate *RUNX2* expression levels, but not sufficiently to cause the CCD-like phenotypic features.

Dentists are often the first clinicians to meet the CCD patients due to dental anomalies, including retention of deciduous teeth, failed eruption of permanent teeth, and supernumerary teeth. We recommended that the dentists familiarize themselves with the phenotypes of CCD and recognize it as a genetic disease.

In summary, we identified a novel 90-kbp deletion (g.103671–193943 del 90273) in the *RUNX2* gene in a Chinese family with CCD and suggested a PCR related detecting method for the large missing fragments. We also summarized the characteristics of all the reported deletions in *RUNX2* and found that the CCD phenotypes were related to the final effects of mutation on protein instead of the length of deletion.

## Figures and Tables

**Table 1:** Gross deletions in *RUNX2* gene.

Number	Description	Predicted Changes in Protein or RNA	Methods	Location	Reference
1	~7.3 Mb incl entire gene + VEGFA + NFKBIE		Microarry	entire areas	[12]
2	~70 kbp incl entire gene		aCGH	entire areas	[17]
3	11.87 kbp incl ex. 8–9		Microarry	PST	[18]
4	nt.220 del173		Sanger sequencing	QA	[19]
5	2.1 Mb, entire gene +~20 additional genes		Q-PCR, aCGH	entire areas	[15]
6	nt.950-971del22	p.Leu317ThrfsX483	Sanger sequencing	PST	[20]
7	500 kbp incl ex. 1–5		Q-PCR, FISH	QA + RHD + NLS + PST	[16]
8	750 kbp incl ex. 1–5		Q-PCR, FISH	QA + RHD + NLS + PST	[16]
9	c.227_306del80	p.Ala76GlyfsX58		QA	[21]
10	9.7 Mb incl. entire gene + CUL7, VEGFA and NFKBIE		aCGH	entire areas	[22]
11	c.230_276del47	p.Ala77ValfsX68	Sanger sequencing	QA	[15]
12	c.718_721del4	p.Ser240CysfsX9	Sanger sequencing	PST	[15]
13	c.879_885del7	p.Ser294ArgfsX12	Sanger sequencing	PST	[15]
14	entire gene		Q-PCR, aCGH	entire areas	[15]
15	ex. 1–3		Q-PCR, MLPA	QA + RHD	[23]
16	ex. 2		MLPA	RHD	[24]
17	125.6 kbp incl ex. 2–6		Sanger sequencing	QA + RHD + NLS + PST	[9]
18	ex. 2–8		Q-PCR	QA + RHD + NLS + PST	[15]
19	ex. 3		Q-PCR	RHD	[15,25]
20	ex. 3–6		Q-PCR	RHD + NLS + PST	[15]
21	ex. 6–8		Q-PCR	PST	[15]
22	ex. 7		Q-PCR	PST	[15]
23	ex. 7–8		Q-PCR	PST	[15]
24	c.241_258del18	p. 84_89del	Sanger sequencing, MLPA	QA	[26,27]
25	c.243-260del18	P.85_90 del	WES, Sanger sequencing	QA	[27,28]
26	~500 kbp incl ex. 1–4 + SUPT3H		Q-PCR CNV analysis by microarrays	QA + RHD + NLS	[29]
27	c.443_454del12insG	p.Val148GlyfsX9	sequencing	RHD	[30]
28	ex. 2–9		unmentioned	QA + RHD + NLS + PST	[30]
29	ex. 6–9		unmentioned	PST	[30]
30	~800 kbp incl entire gene		aCGH	entire areas	[31]
31	~90 kbp incl ex. 4–7	p.Asn294LlefsX1	WES, PCR, Sanger sequencing	RHD + NLS + PST	This paper

incl: include; del: deletion mutation; ins: insertion mutation; ex: exon; fs: frameshift; aCGH: array comparative genomic hybridization; FISH: fluorescence in situ hybridization analysis; MLPA: multiplex ligation-dependent probe amplification; WES: whole exome sequencing; CNV: subsequent copy number variation; QA: glutamine–alanine repeats domain; RHD: Runt homologous domain; NLS: nuclear localization signal; PST: proline/serine/threonine rich region.

## Data Availability

Research data not shared.
